# Depression, anxiety, stress and other mental health conditions of personnel undergoing hospital isolation during the COVID-19 pandemic and influencing factors

**DOI:** 10.12669/pjms.40.3.7511

**Published:** 2024

**Authors:** Baoyan Wang, Lei Li, Jing Liu, Bo Zhan

**Affiliations:** 1 Baoyan Wang, Department of Infectious Disease, Baoding No.1 Hospital, Baoding 071000, Hebei, China; 2 Lei Li, Department of Rheumatology and Immunology, Baoding No.1 Hospital, Baoding 071000, Hebei, China; 3 Jing Liu, Department of Rheumatology, Baoding No.1 Central Hospital, Baoding 071000, Hebei, China; 4 Bo Zhan, Department of Infectious Disease, Baoding No.1 Hospital, Baoding 071000, Hebei, China

**Keywords:** COVID-19, Personnel undergoing hospital isolation, Anxiety, Depression, Stress, Mental health

## Abstract

**Objective:**

To understand the depression, anxiety, stress and other mental health conditions of personnel undergoing hospital isolation during the COVID-19 pandemic and the influencing factors.

**Methods:**

This was retrospective study. A total of 120 personnel undergoing Baoding No.1 Hospital isolation who completed the questionnaires were included from June 10, 2021 to February 07, 2022. The Patient Health Questionnaire-9(PHQ-9), the Generalized Anxiety Scale (GAD7) and psychological stress measurement table (PSTR) were used for psychological problem screening for personnel undergoing hospital isolation.

**Results:**

The incidence of depression was the lowest, while that of stress was the highest. The difference in the incidence of depression, anxiety and stress among personnel undergoing hospital isolation with different gender, age, income statuses, marital statuses and attitude towards isolation was statistically significant (p< 0.05), while the difference in the incidence of these problems among personnel with different degree of education was not statistically significant(p> 0.05). Multivariate Logistic regression analysis showed that age, gender, marital status, economic status and attitude towards isolation are factors associated with stress. Economic status and attitude towards isolation are factors associated with depression. A high economic level is a protective factor against depression, while a negative attitude is a risk factor for depression.

**Conclusion:**

During the COVID-19 pandemic, anxiety, depression and stress increased to different extents in personnel undergoing hospital isolation, especially in females with poor economic conditions and poor attitudes towards isolation. Therefore, necessary psychological counseling and social support should be provided to these people.

## INTRODUCTION

COVID-19 is an emerging infectious disease with such epidemiologic features as a complex transmission route, strong infectivity and general susceptibility caused by a novel coronavirus of β coronavirus genus.^1^ The World Health Organization (WHO) classified the COVID-19 pandemic as a “global pandemic” in 2020.^2^ It has diversified transmission routes, high incidence and strong infectivity. Its clinical manifestations are fever, respiratory symptoms, imaging features of pneumonia, normal or decreased white blood cell count or reduced lymphocyte count at the early stage of the disease.^3^ The early mortality is high. Although the mortality gradually decreases over time, it still has certain mortality for infected people.^4^ At present, an isolation regime, including hospital isolation and centralized isolation for medical observation, is implemented on close contacts in many countries and regions.

During the COVID-19 pandemic, most patients in isolation wards show irritability, panic, anger and other emotions, which impairs the body’s immune system and may cause serious impairment of the entire psychological system. Moreover, personnel undergoing hospital isolation are also suffering the consequences of negative emotions, such as depression, anxiety and stress.^5^ Serious psychological problems may also affect physical health. Therefore, it is necessary to explore the negative emotions of personnel undergoing hospital isolation during COVID-19 and provide early intervention. This study was intended to survey the distribution characteristics of the depression, anxiety and stress of personnel undergoing hospital isolation during the COVID-19 pandemic to provide a basis for regulating the physical and mental health of isolated personnel and relevant psychological intervention.

## METHODS

This was retrospective study. A total of 120 personnel undergoing Baoding No.1 Hospital isolation were included in this survey from June 10, 2021 to February 07, 2022. Patient data including demographic data and diagnosis of COVID-19 were retrieved from electronic medical record systems undergoing Baoding No.1 Hospital isolation. All isolated personnel received the psychological problem screening and psychological assessment within 24 hour after the beginning of the isolation, (Table I).

**Table-I:** General Data of Study Subjects (*χ̅*±S) n=120.

Item	Number of cases	Proportion
***Gender***		
Male	90	75.3%
Female	30	24.7%
***Age (y)***		
18~30	38	31.6%
30~60	57	47.4%
60~70	25	21%
***Degree of education***		
Middle school	16	13.8%
University	67	55.6%
Above university	37	30.6%
***Income***		
High	56	46.6%
Moderate	44	37%
Low	20	16.4%
***Attitude towards isolation***		
Cooperative	76	63.2%
Complaining	44	36.8%
***Marital status***		
Married	80	67%
Unmarried	29	24.2%
Divorced	11	8.8%

### Ethical Approval

The study was approved by the Institutional Ethics Committee of Baoding No.1 Hospital (No.:2022022207; Date: February 22, 2022), and written informed consent was obtained from all participants.

### Inclusion criteria:

- People who are above 18 and below 70 in age;
- People who are informed and willing to participate in this survey.

### Exclusion criteria:

- People who have dementia, aphasia, or cognitive impairment or are unable to cooperate with the study.
- Patients with serious underlying disease or infectious disease.
- The general data questionnaire mainly covers name, gender, age, degree of education, marital status, family economic level and attitude towards isolation.

### The Patient Health Questionnaire-9 (PHQ-9)

It is composed of nine items and adopts Likert4 grade scoring. The answer and score for each item are as follows: not at all (0), several days (1), more than one week (2) and almost every day (3). The total score is 27 points. The higher the score, the greater the likelihood of depression. Based on the score, the degree of depression can be divided into mild depression (6-9 points), moderate depression (10-14 points), severe depression (15-21 points), and extremely severe depression (22-27 points).^6^

### The Generalized Anxiety Scale (GAD-7)

It is composed of seven items and also adopts Likert4 grade scoring, specifically as follows: not at all (0), occasionally (1 point), frequently (2 points), almost every day (3 points). According to the score, the degree of anxiety is divided into mild anxiety (5~9 points), moderate anxiety (10~14 points) and severe anxiety (above 15 points).^7^ The PSTR: It includes 50 items, each of which is scored 0~4. The higher the score, the greater the stress. A score of 43-65 points, lower than 43 points and higher than 65 points indicates moderate stress, small stress and great stress, respectively.^8^ All the questionnaires were given to the participants for investigation, and then collected in about five minutes.

### Statistical analysis

The software SPSS 20.0 was used for the statistical analysis of all data. The measurement data were expressed as (*χ̅*±S). The sample size was estimated by 95% confidence interval. Independent samples t-test and ANOVA were used for intergroup data analysis, while Logistic regression analysis was used for the analysis of relevant risk factors. P<0.05 was considered statistically significant.

## RESULTS

The incidence of depression, anxiety and stress in personnel undergoing hospital isolation was 21.6%, 31.4% and 33.8%, respectively. The incidence of depression was the lowest, while that of stress was the highest, (Table-II).

**Table-II:** Incidence of Depression, Anxiety and Stress in Personnel Undergoing Hospital Isolation (*χ̅*±S) n=120.

Index	Mild (number of cases)	Moderate (number of cases)	Severe (number of cases)	Total	Incidence
Depression	14	7	5	26	21.6%
Stress	22	12	7	41	33.8%
Anxiety	23	8	6	37	31.4%

The analysis of depression (PHQ-9), anxiety (GAD-7) and stress (PSTR) in personnel undergoing hospital isolation with different characteristics indicated that the difference in the score in personnel undergoing hospital isolation with different gender, age, income status, marital status and attitude towards isolation was statistically significant (p< 0.05), while the difference in the score in those with different degree of education was not statistically significant(p> 0.05), (Table-III).

**Table-III:** PHQ-9, GAD-7 and PSTR Analysis for Personnel Undergoing Hospital Isolation (*χ̅*±S).

Item	PHQ-9			GAD-7			PSTR		

	Score	t/F	p	Score	t/F	p	Score	t/F	p
Gender*		14.09	0.00		4.23	0.00		8.06	0.00
Male	4.36±1.24			7.13±2.97			52.67±6.82		
Female	6.73±2.44			8.47±3.25			58.24±6.04		
Age (y)*		7.45	0.00		3.66	0.00		2.08	0.00
18~30	4.63±1.79			6.90±2.74			54.13±5.88		
30~60	6.58±2.03			7.89±3.07			56.47±6.06		
60~70	5.66±3.01			7.37±2.72			57.64±5.47		
Degree of education		0.49	0.62		0.09	0.93		0.11	0.91
Middle school	4.73±1.48			7.43±2.21			55.89±6.00		
University	4.70±1.32			7.47±2.18			55.76±6.12		
Above university	4.77±1.49			7.45±2.07			55.83±6.24		
Income*		4.18	0.00		2.28	0.02		8.79	0.00
High	3.17±1.28			5.62±2.04			42.75±6.74		
Moderate	3.76±1.43			6.08±2.37			48.79±5.80		
Low	4.35±1.33			6.53±2.15			56.75±7.06		
Attitude towards isolation*		11.83	0.00		10.05	0.00		10.67	0.00
Cooperative	3.46±1.67			5.17±2.24			48.57±8.43		
Complaining	5.78±2.43			7.06±2.47			57.72±7.59		
Marital status*		7.46	0.00		8.77	0.00		11.78	0.00
Married	4.75±1.20			6.83±2.28			62.81±7.54		
Unmarried	3.12±1.32			5.30±2.10			48.79±7.28		
Divorced	3.63±1.54			6.21±1.87			52.63±7.68		

* p< 0.05.

With age, gender, marital status, economic status and attitude towards isolation as independent variables, multivariate logistic regression analysis was performed after assignment, α _input_=0.05, α_output_=0.10, the results showed that: age (p= 0.000), gender (p= 0.000), marital status (p= 0.001), economic status (p= 0.000), attitude towards isolation (p= 0.000) are factors associated with stress. High economic level is a protective factor against stress, while gender, female, married and divorced, and negative attitude are risk factors for stress, (Table-IV).

**Table-IV:** Multivariate Logistic Regression Analysis of Risk Factors for Psychological Stress in Personnel Undergoing Hospital Isolation n=120.

Correlated variable	β value	SE value	Waldχ^2^	P value	OR value	95.0% CI
Age (reference group = 18~30)	2.728	0.936	8.421	0.000	14.535	13.581~15.827
Gender (reference group = male)	2.438	0.761	7.573	0.000	6.974	5.486~8.540
Marital status (reference group = unmarried)	3.572	0.625	8.966	0.001	5.351	4.763~7.644
Economic level (reference group = low)	-3.718	0.873	6.751	0.000	5.408	4.378~6.703
Attitude towards isolation (reference group = cooperative)	2.674	0.628	7.783	0.000	11.876	10.582~12.497

p< 0.05.

With age, gender, marital status, economic status and attitude towards isolation as independent variables, multivariate logistic regression analysis was performed after assignment, α _input_= 0. 05, α_output_= 0.10, the results showed that: gender (p= 0.000), economic status (p= 0.000), attitude towards isolation (p= 0.000) are factors associated with anxiety. High economic level is a protective factor against anxiety, while female and negative attitudes are risk factors for anxiety, (Table-V).

**Table-V:** Multivariate Logistic Regression Analysis of Risk Factors for Anxiety in Personnel Undergoing Hospital Isolation n= 120.

Correlated variable	β value	SE value	Waldχ^2^	P value	OR value	95.0% CI
Age (reference group = 18~30)	0.198	0.175	1.162	0.281	1.238	0.856~1.821
Gender (reference group = male) *	0.427	0.797	13.173	0.000	1.534	1.254~1.876
Marital status (reference group = unmarried)	0.398	0.417	0.980	0.347	0.761	0.278~1.380
Economic level (reference group = low) *	1.183	0.124	7.021	0.000	3.237	2.620~4.089
Attitude towards isolation (reference group = cooperative) *	1.183	0.307	13.982	0.000	3.723	2.681~5.070

* p< 0.05.

With age, gender, marital status, economic status and attitude towards isolation as independent variables, multivariate logistic regression analysis was performed after assignment, α _input_=0. 05, α_output_=0.10, the results showed that: economic status (p= 0.000) and attitude towards isolation (p=0.000) are factors associated with depression. High economic level is a protective factor against depression, while a negative attitude is a risk factor for depression, (Table-VI).

**Table-VI:** Multivariate Logistic Regression Analysis of Risk Factors for Depression in Personnel Undergoing Hospital Isolation n= 120.

Correlated variable	β value	SE value	Waldχ^2^	P value	OR value	95.0% CI
Age (reference group = 18~30)	0.018	0.179	0.554	0.421	0.920	0.826~1.157
Gender (reference group = male)	1.231	0.539	5.247	0.073	1.325	1.107~1.563
Marital status (reference group = unmarried)	0.043	0.327	0.024	0.875	1.033	0.764~1.259
Economic level (reference group = low) *	1.221	0.108	9.832	0.003	3.378	2.720~4.187
Attitude towards isolation (reference group = cooperative) *	1.146	0.297	13.047	0.002	3.153	1.761~5.632

* p< 0.05.

## DISCUSSION

According to the results of this study, the incidence of depression, anxiety and stress in personnel undergoing hospital isolation was 21.6%, 31.4% and 33.8%, respectively. Age, gender, marital status, economic status and attitude toward isolation are factors associated with stress. A high economic level is a protective factor against stress, while gender, female, married and divorced, and negative attitude are risk factors for stress. Gender, economic status and attitude towards isolation are factors associated with anxiety. Economic status and attitude towards isolation are factors associated with depression.

Isolated medical observation is a measure to isolate people who may have been exposed to infection sources and restrict their range of activity, so as to reduce the risk of infecting others and avoid the spread of infectious diseases in the population. Studies have shown that^9^, in public health emergencies, the isolated personnel often show different degrees of anxiety, depression, insomnia and other psychological symptoms and that some psychological problems are persistent and complex.^10^ Therefore, the influence of negative emotions of isolated personnel should be reduced as far as possible while effectively coping with the COVID-19 epidemic by means of isolation.

Anxiety, depression and stress are people’s subjective emotional experiences. Anxiety is mainly manifested as nervousness, worry, restlessness, and autonomic symptoms without a clear object.^11^ Depression is clinically manifested as significant and persistent low mood, which does not fit with the situation. Anxiety, depression and somatic symptoms may exist separately or in combination, or occur with physical disease.^12^ Both anxiety and depression affect patients’ social role and quality of life(QOL), resulting in physical, psychological, cognitive and behavioral abnormalities. Due to the lack of specific treatment, COVID-19 has strong infectivity and an increasing number of confirmed patients. Besides, patients are faced with unprecedented fear of death. Furthermore, the isolation mode makes patients fearful, lonely, and helpless.^13^ During the COVID-19 pandemic, patients undergoing hospital isolation suffer from intensified mental exhaustion, burnout, fear, depression, anxiety, insomnia and psychological stress. Such stress may exert a negative impact on the safety of patients and isolated personnel.^14^

The COVID-19 outbreak is a stressful life event that cannot be controlled. Isolating measures disrupt daily life^15^, it is important to evaluate and treat the negative emotions of the isolated personnel during the COVID-19 pandemic. This study proved that, age, gender, marital status, economic status and attitude towards isolation are main factors affecting the negative emotions of personnel undergoing hospital isolation. Therefore, it is crucial to consider these factors while planning effective intervention strategies to reduce the risk of poor mental health.^16^ Among isolated personnel, females are more susceptible to anxiety, depression and stress. Alemanno et al.^17^ included 87 COVID-19 patients.

The results of MMSE, MoCA, Hamilton Depression Rating Scale and FIM showed that all patients need long-term psychological support and treatment to prevent the occurrence and aggravation of psychological diseases. Alkhamees et al.^18^ believed that nearly a quarter of the sampled general population suffered from moderate to severe psychological effects throughout the COVID-19 outbreak in Saudi Arabia. Taking specific preventive measures can protect individuals’ mental health. Public mental health strategies should be implemented in the early days of the COVID-19 outbreak. According to Wang et al.^19^, one third of adults among general population suffer from COVID-19-associated psychological disturbance. Joint efforts are urgently needed to intervene with high-risk groups. With the further spread of COVID-19 pandemic, it is particularly important to develop psychological intervention strategies and provide continuous intervention^20^.

### Limitations

The sample selection and sample size are limited due to limited time. Long time, extensive and in-depth surveys and studies based on large sample sizes will be carried out in the future. In addition, personnel undergoing hospital isolation and centralized isolation will be grouped for comparison. The purpose is to identify more affecting factors to provide more refined or targeted medical interventions to isolated personnel due to the pandemic.

## CONCLUSION

During the COVID-19 pandemic, anxiety, depression and stress increased to different extents in personnel undergoing hospital isolation, especially in females with poor economic conditions and poor attitudes towards isolation. Therefore, relevant personnel should provide necessary psychological counseling and social support to these people. In addition, different counseling methods should be taken according to different psychological problems of different objects, so as to timely solve the psychological problems of personnel undergoing hospital isolation.

### Authors’ Contributions:

**BW** and **LL:** Designed this study, prepared this manuscript, are responsible and accountable for the accuracy and integrity of the work.

**JL:** Collected and analyzed clinical data.

**BZ:** Participated in acquisition, analysis, or interpretation of data and draft the manuscript.

All authors read and approved the final manuscript.
